# Mandatory Vaccination during the Period of a Pandemic: Legal and Ethical Considerations in Europe †

**DOI:** 10.3390/biotech10040029

**Published:** 2021-12-13

**Authors:** Fereniki Panagopoulou

**Affiliations:** Department of Public Administration, School of Economy and Public Administration, Panteion University of Social & Political Sciences, 136, Syggrou Ave., 176 71 Athens, Greece; fereniki@panteion.gr

**Keywords:** vaccination, legal framework, fundamental rights, pandemic, self-determination, vaccine hesitancy

## Abstract

The present study explores the pressing matter of mandatory vaccination in Europe from an ethical–constitutional perspective. To start with, it considers the bases of the concerns that have been raised to date, as well as those of the documented opposition. This is followed by an analysis of the applicable European legal framework and a discussion on mandatory vaccination in the workplace, education and the leisure industry, before outlining the conclusions reached. The position taken by this paper is that as long as certain conditions are met, mandatory vaccination does not violate fundamental rights. On the contrary, provided that the principle of proportionality is satisfied, mandatory vaccination as a form of medical intervention constitutes a manifestation of the obligation on the part of the state to protect the fundamental rights to life and health.

## 1. Introduction

The recent coronavirus pandemic and the immense global irregularity that followed it brought a number of ethical–constitutional issues to the table, which shall remain compelling even when we manage to reach the end of this nightmare, thanks to the contribution of science. The initial concern that was raised related to the prioritization of the delivery of the vaccine to specific population groups and countries. Rich countries hastened to over-fulfil their needs by purchasing more vaccines than needed, exhibiting rampant chauvinism [1] and indifference towards the needs of other countries and the quest to achieve global immunity. The matter of prioritization is directly linked to the ancillary issue of mandatory vaccination, as the latter cannot be conceived without vaccine availability. Indeed, it is an issue that has beset humanity ever since the first emergence of vaccines, given that from their invention and up to this day, it continues to cause heated debate. Those in favor of mandatory vaccination put forward the protection of public health as the primary objective and value in the case at hand, whilst those opposing this notion emphasize their right to self-determination, as well as their reservations concerning the side effects of vaccines.

## 2. The Sources of Vaccine Hesitancy

The history of the anti-vaccination movement is certainly not unclouded, nor is the movement a product of the recent pandemic. In 1885, for instance, a protest took place in Montreal against the law that made vaccination against smallpox mandatory [2]. A strong anti-vaccination movement also emerged towards the end of the 1990s, following a non-substantiated report that was published in 1998, which implied a causal link between the MMR (measles, mumps and rubella) vaccine to autism. This study was withdrawn shortly afterwards and, in fact, the researcher who conducted it lost his licence to practice medicine. Nevertheless, it had a very strong impact on the pledge in favour of vaccination and the sense of mistrust that was created around it.

Reluctance and anti-vaccination movements usually stem from insecurity and mistrust surrounding pharmaceutical companies, which have not, at times, shown the best of conduct, given their subjection to considerable economic interests [3]. Mistrust is also expressed against science itself (in fact, to be exact, with regard to its degree of independence), often precisely because of the existence of scientific controversies that end up dividing the public. The plurality of dialogue, along with the uncontrollable dissemination of information, has often led to the creation of a cacophony. In addition to the above, the contribution of false news to this sense of mistrust has also been considerable.

Furthermore, there have been more than a few reactions owed to the side effects of vaccinations, based on the findings of experts, personal accounts, pleas in favour of the best interests of the child, the need to return to nature and natural methods of disease prevention, the value of free and informed choice, the fight against big interests that pharmaceutical companies seem to be serving and so on [4]. The agony putting populations under surveillance through vaccination [5], which is linked to all kinds of unsubstantiated conspiracy theories, is also added to the above concerns.

Some other contributors to COVID-19 vaccine hesitancy among health care professionals include safety concerns, doubts about effectiveness (in terms of individual protection and/or reduction in transmission) and perceived low risks of infection among health care professionals who do not treat patients with COVID-19 [6].

We should also not overlook certain social, cultural and political considerations [7]. Anti-vaccination sentiments are also partly linked to matters of conscientious objection. Indeed, the 1898 Vaccination Act in England allowed persons to opt out of vaccination for moral reasons, allowing exemption for parents who wished to obtain a certificate of exemption in order not to have their children vaccinated.

## 3. European Legal Framework

### 3.1. Article 8 ECHR

Vaccination constitutes a medical intervention. A medical intervention against the subject’s will contravenes his or her right to physical integrity, which is a manifestation of the right to private life pursuant to Article 8 ECHR [8,9,10,11,12]. The European Court of Human Rights has held that the imposition of medical treatment without the consent of a mentally competent adult patient, even where the refusal to accept a particular treatment might lead to a fatal outcome, impinges on the right to physical integrity [9,13,14]. Accordingly, the forced administration of medication constitutes interference with the right to private life [8]. Vaccination without the consent of the subject constitutes, without doubt, a violation of Article 8 ECHR, which protects private life and, by extension, the physical and psychological integrity, as well as the autonomy of the subject [10,15,16]. An exception to this could be mandatory vaccination during the period of a pandemic, so as to protect third parties [17] (p. 371).

The European Commission of Human Rights (ECHR) had already underlined in 1984 that a requirement to undergo medical treatment or a vaccination, on pain of a penalty, may amount to interference with the right to respect for private life [18]. This position was subsequently verified by the ECtHR, which held that mandatory vaccination—as an involuntary medical treatment—amounts to an interference with the right to respect for one’s private life, which includes a person’s physical and psychological integrity, as guaranteed by Article 8§1 ECHR [19]. The Court’s view was that what has to be determined is whether such interference may be justified on the basis of Article 8§2 ECHR, noting that such interferences were expressly provided by law and pursued the legitimate aim of protection of public health. Therefore, what to be assessed was whether this interference was necessary in a democratic society. The Court’s conclusion in this case was that the interference with the applicant’s physical integrity could be said to be justified in light of public health considerations and the necessity to control the spreading of infectious diseases in the region. Indeed, based on the domestic court’s findings, the medical staff had checked the subject’s suitability for vaccination prior to carrying out the vaccination, suggesting that necessary precautions had been taken to ensure that the medical intervention would not be to the applicant’s detriment to the extent that would upset the balance of interests between the applicant’s personal integrity and public health protection. Consequently, it would appear that when mandatory vaccination can be justified in view of protecting public health and it is aimed specifically at preventing the spreading of infectious diseases, whilst not being medically contraindicated in the individual case under consideration, it is in compliance with 8§2 ECHR, as it constitutes a limitation that is necessary in a democratic society [19].

In a similar vein, the European Commission of Human Rights has also held that compulsory vaccination of children against hepatitis B may be regarded as an interference mandated by a legitimate aim that falls under Article 8§2 ECHR, namely the need to protect public health, as well as that of the persons concerned [20]. Therefore, the Commission found that this intervention was justified and went on to examine whether the interference in the applicant’s private life was also “necessary in a democratic society”.

According to the Court, the right to free choice and self-determination may be curtailed when doing so is necessary for the protection of third parties, precisely as in the case of mandatory vaccination in the middle of a pandemic [18]. In the case of the Jehovah’s Witnesses of Moscow, the ECtHR held that free choice and self-determination were themselves fundamental constituents of life and that, absent any indication of the need to protect third parties—for example, mandatory vaccination during an epidemic, the State must abstain from interfering with the individual freedom of choice in the sphere of health care, for such interference can only lessen and not enhance the value of life [18]. Consequently, the Court held that, as a matter of principle, the right to life may be limited to protect third parties.

The judgment of the ECtHR, following its referral from the First Section of the Court to the Grand Chamber, in the case concerning mandatory vaccination in the Czech Republic, is still hot off the press [21]. In the written comments submitted before the Court in relation to this case, the European Centre of Law and Justice underlined the fact that Europe remains quite divided on this matter. The Court’s ruling was that mandatory vaccination does not violate Article 8 ECHR, thus paving the way for the imposition of similar measures for the protection of public health against the COVID-19 pandemic, insofar as they could be deemed necessary [22]. In this case, the Court found, with a majority of 16-1, that the mandatory vaccination of children, as a medical act that lacks consent, does fall under the realm of application of Article 8 ECHR but it does not, ultimately, violate it. This is applicable provided vaccination aims at protecting individual and public health, as well as the rights of third parties, such as persons who are unable to undergo vaccination on health grounds and thus remain at constant risk until herd immunity is achieved. As remarked by the Court, a duty of social solidarity is owed to these people. It should also not be overlooked that Articles 2 and 8 impose a positive obligation upon states for the protection of the life and health of their citizens, particularly in countries where relevant resources are available [23]. At the same time, it was also noted that member states do not share a common position on vaccination. Therefore, in view of the lack of consensus between the parties to the ECHR, it is essentially up to the states themselves, given the wide margin of appreciation they possess, to consider and decide the extent to which scientific data support mandatory vaccination. The assessment of experts and relevant medical authorities on this matter is also crucial, as it provides the basis for ascertaining the existence of a pressing social need. These elements, on a national and international level, were also stressed by the Czech Constitutional Court when it ruled in favor of the constitutionality of the measure of mandatory vaccination. Another parameter that the Court considered was the best interests of the child, which are served, without a doubt, by the Czech Republic’s vaccination policy. Bearing in mind the above, the legislation of mandatory vaccination in the Czech Republic is mandated by the need for the protection of the health and life of its citizens. With reference to the proportionality of the measure, the Court did not examine whether the Czech Republic could have adopted a different policy of elective vaccination, as is the case in other European countries; it confined its examination to whether the specific measure in question, considered in the context of the circumstances of its application, could be deemed proportionate and, in view of this, whether it is a restriction that is “necessary in a democratic society” in accordance with Article 8§2 ECHR. At the instance in question, the Court also took into consideration the fact that the law does not provide for the possibility of physical coercion but only for other sanctions, such as precluding registration in preschool facilities and potentially (low) penalties.

The case law of the ECtHR has held that although vaccination does constitute an interference, it is not necessarily also a violation of the right to private life, as said interference may be justified. In order to do so, three conditions must be met, starting with that the interference should be provided by legislation which, in accordance with the rule of law, must be easily accessible and foreseeable. It should also serve a lawful purpose, such as, in the present case, the protection of public health, as well as the rights of third parties, meaning our fellow human beings, against contracting the virus from us. Lastly, the interference must be proportionate to the legitimate aim pursued. The principle of proportionality is satisfied to the extent that the measure adopted takes into consideration the state of the subject, meaning that potential serious contra-indications to the vaccination of the person in question should not be overlooked. Under these circumstances, a vaccination campaign that obliges a person to yield to the interests of the general public and not put the health of fellow human beings at risk, provided that his or her own life is also not in danger, does not overstep the margin of appreciation vested in the state. The ECtHR recognizes a margin of appreciation on the part of states with regard to the necessity of vaccination, the application of sanctions, and the imposition of penalties. The Court has held that reasonable sanctions in the event of non-justified refusal to be vaccinated, such as restrictions to travel and movement, quarantine or the imposition of a fine, do not appear to be incompatible with the European Convention on Human Rights, considering the relevant case law to date [24]. Assuming that a measure has been enacted on the basis of an accessible and foreseeable legal provision [25], the assessment of its necessity in the context of a democratic society would be very interesting and of notable importance.

According to a recent ECHR press release, the Court rejected the requests for interim measures submitted by 672 full-time and voluntary members of the French fire service against mandatory vaccination for the management of COVID-19 in France. It considered that those requests lay outside the scope of Rule 39 of the Rules of Court, according to which such requests may only be granted on an exceptional basis, when the applicants would otherwise face a “real risk of irreversible harm”. The applicant fire fighters had invoked ECHR provisions on the “right to life” and the “right to respect of private and family life”, seeking the “suspension of the requirement to be vaccinated”, as set out in the Law of 5 August 2021 on the management of the public health crisis. In the alternative, they requested the suspension of the provisions “prohibiting persons who have failed to comply with the requirement to be vaccinated from exercising their occupation” and those “interrupting the payment of salaries to persons who have failed to comply with the requirement to be vaccinated”. The relevant decision on the part of the ECtHR does not prejudge, according to its rules, any “subsequent decisions on the admissibility or merits of the case”.

Lastly, according to the relevant ECHR press release [26], on 2 September 2021, the European Court of Human Rights received two applications against Greece, lodged by 30 health professionals who work independently or in public health institutions. The applicants complained about the provisions of Section 206 of Law No. 4820/2021, which impose mandatory vaccination in the health-sector. They requested that the Court apply interim measures (Rule 39 of the Rules of Court), and that it suspend immediately the application of the law in question. On 7 September 2021, the Court decided to reject the requests for interim measures, holding that they were outside the scope of Rule 39 (interim measures). The Court pointed out that measures under Rule 39 of the Rules of Court are decided in connection with proceedings before the Court, without prejudging any subsequent decisions on the admissibility or merits of the case. The Court grants such requests only on an exceptional basis, when the applicants would otherwise face a real risk of irreversible harm. It is also noted that the applications in question are currently pending before the Court [27,28].

### 3.2. The Oviedo Convention (The Convention for the Protection of Human Rights and Dignity of the Human Being with Regard to the Application of Biology and Medicine: Convention on Human Rights and Biomedicine)

Articles 5 et seq. of the Oviedo Convention, which has been ratified in the Greek legal order pursuant to Law No. 2619/1998, establish the principle of informed consent. According to Article 5 of the Convention, “An intervention in the health field may only be carried out after the person concerned has given free and informed consent to it. This person shall be given appropriate information beforehand as to the purpose and nature of the intervention, as well as on its consequences and risks. The person concerned may freely withdraw consent at any time”.

Bearing in mind the above, a medical act is not permissible without the informed consent of the patient. Nevertheless, the provision in question is not absolute. Article 26 of the Convention sets the protection of public health, among others, as a restriction to the exercise of rights provided for by the Convention. Indeed, Article 26§2 of the Convention does not exempt the right to informed consent from the related restriction. This is expressly stipulated in paragraph 39 of the explanatory report to the Convention [29].

It is a fact that the wording of Article 26 of the Oviedo Convention evokes the limitations contained in Article 8 of the ECHR. With consideration to the preparatory work on the Convention, it follows that the case of mandatory vaccination is covered by the provision on public health under Article 26 of the Convention [30] (p. 25). Consent to the medical act in this case has an impact not only on the subject of the decision, but also puts in danger the health of others [31]. The notion of health must also be interpreted broadly and covers even the mandatory isolation of a patient, without his or her consent, in the event of a serious infectious disease [29]. In view of the above, mandatory vaccination with the aim of protecting public health does not give rise to an issue of violation of the Oviedo Convention, as the Convention itself provides the necessary limitations to the principle of informed consent [32]. With regard to this point, it must also be taken into consideration that the wording and overall structure of Article 26, as expressly transpiring from the preparatory work on the Convention and especially the explanatory report that accompanies it, make reference to the equivalent limitations on the principle of informed consent that are set out in Article 8§2 of the ECHR, as also interpreted in the relevant case law of the ECtHR [29].

## 4. Is the Measure of Mandatory Vaccination Proportionate?

On a first level, it must be explored whether the measure of mandatory vaccination is appropriate for addressing the pandemic. The transmission of the disease may only be substantially reduced if the largest part of the population is vaccinated—meaning about 75%—so as to achieve herd immunity. On the contrary, if only part of the population is vaccinated—for example, 50%—then the spreading of the disease will not be curtailed. Therefore, the measure is deemed appropriate at first glance. At this point we must also stress the lack of studies on the transmission of the disease by vaccinated persons. However, even if transmission continues to take place by vaccinated persons, the benefit offered to public health by vaccination should not be underestimated. This is because vaccinated persons, even if they contract the virus, will not, as a rule, have severe symptoms and will not occupy hospital beds in Intensive Care Units to the detriment of other patients who need them [33]. In addition to the above, vaccinated carriers of COVID-19 appear to have a lower viral load, which means that they do not transmit the virus to the same extent as unvaccinated persons.

On a second level, the question whether this measure is necessary must be assessed. In this context, it is examined whether other milder, but equally effective measures, are available. Such a measure could be an information campaign on vaccination. Therefore, in terms of necessity, it is imperative to have an information campaign in place. Establishing mandatory vaccination requires the exhaustion of the effort to persuade people about vaccination. This exhaustion relates to other delays in the establishment of mandatory vaccination, which are consequential to the principle of proportionality. From the point of view of state power, the center of gravity must be the information campaign in favor of vaccination, as human fear is overcome with information—not coercion [33]. If information fails to deliver the anticipated outcome, which is what has happened, mandatory vaccination must follow.

If the largest part of the population is voluntarily vaccinated with the available vaccines, there is no issue of establishing mandatory vaccination for the entire population. Therefore, if the required percentage for attaining herd immunity is achieved anyway through elective vaccination, mandatory vaccination is not deemed a necessary measure.

A milder measure than mass mandatory vaccination is targeted mandatory vaccination for specific population groups, such as health professionals, educators, people working in camps or key staff in certain sectors of activity, such as police officers and fire fighters. Furthermore, firing unvaccinated employees across the board may, at face value, be seen as non-necessary measure, given that milder measures are available, such as, for example, putting such persons on suspension, etc. [34].

Other legal consequences, such as prohibiting access to crowded areas, including public transport, game fields, theaters, cinemas and health regulated establishments, must also be examined under the light of necessity. Is a negative test result certificate resulting from a rapid self-test, for example, equally effective as a vaccination certificate? The chances of an erroneous positive diagnosis in the case of a rapid self-test or even a false negative result, in view of the fact that positivity is diagnosed a few days following transmission, should also be considered and reflected upon. Bearing in mind the above, such prohibitions are deemed necessary when there exist no other, equally effective solutions [35] (p. 25).

Notwithstanding the above, allowing entry solely on the basis of a specific certificate should not have universal application. In this context, the absolute prohibition of entry to public transport for unvaccinated persons would likely be considered unconstitutional, as such a measure would restrict their commute to work and their place of residence. An alternative possibility would be the obligation to present a negative test result [33], conducted at the expense of the person being examined, even if these do not guarantee the absolute accuracy of the test result. The issue of public transport is one that must be approached with great caution. An absolute ban from using public transport in the case of unvaccinated individuals is deemed unconstitutional, as this could mean that these persons would be entirely unable to travel to their work or visit their family and friends, whilst also leading to their total alienation from the social fabric [33].

With reference to the prohibition of entry to schools, this is a matter that needs to be assessed based on the data related to the transmission of the virus between pupils. If the spreading of the transmissibility of the virus is not considerable, other measures should be considered. What is of utmost importance, however, is the need to conduct wide-ranging epidemiological studies that will prove the suitability of vaccines for pupils.

What appears to be more challenging is the examination of proportionality in a narrow sense. With reference to this point, what needs to be considered is whether the intervention to the free development of personality, the protection of privacy and personal data, the right to education and the principle of equality takes place in harmonious balance with the need to protect public health. At this stage, the strict correspondence between the advantages and disadvantages of mandatory vaccination and the mathematical (but not arithmetic) cost and benefit balance of this measure is sought; in other words, what needs to be determined is the balance between different interests or the practical harmony between the measure, its aim and the actual situation [36] (p. 889 et seq. and pp. 81–82). In this respect, it is crucial to opt for the solution which, in the context of the in concreto balancing exercise, takes into consideration the entire sum of the possible or concurrently conflicting interests, while, at the same time as serving the remaining public interests, it preserves intact the core of the rights to the protection of health, the development of personality, personal data, equality, education and the development of entrepreneurial activity [36] (p. 889 et seq. and p. 82).

Under ordinary circumstances, mandatory vaccination conflicts with the right to self-determination [37]. A person cannot be subjected to something when it is possible that it may bring about side-effects, even if they are entirely isolated. The rule is that vaccination is recommended, and in some cases highly so, but it is not imposed; therefore, non-vaccination cannot be linked to adverse consequences involving exclusion from social life [38] (p. 359 et seq.).

Nevertheless, should vaccination be deemed medically required for the immediate protection of public health, and provided this is assessed on the basis of substantiated studies from the medical communities, it may be made mandatory in exceptional circumstances, particularly in relation to specific population groups and not to the entire population.

For the measure of mandatory vaccination to be proportionate, the following must apply:

Firstly, mandatory vaccination must be justified by an overriding reason of public interest and the vaccine must be the decisive tool of serving said public interest [39] (p. 248). Secondly, vaccination should not be physically imposed. We cannot have a doctor chasing us with a syringe to administer the vaccine, as this would violate the value of the human person. Notwithstanding the above, non-vaccination may be linked to administrative penalties, such as monetary fines or prohibition of access to certain specified public areas or services in crowded places [40] (p. 250). Thirdly, vaccination cannot be imposed if it is not accessible to the entire population. This means that it cannot be made mandatory if it is no available. If vaccination is not accessible to everyone, non-vaccination cannot entail negative consequences. Next, mandatory vaccination mandatory must be preceded by the conduction of wide-ranging epidemiological studies showing that it does not cause negative side effects beyond those expected. In view of the lack of such studies when it comes to children, vaccination cannot be made mandatory to attend lessons and non-vaccination cannot constitute a reason for not allowing pupils into schools. In other words, the safety and effectiveness of the vaccine should be guaranteed [39]. Furthermore, vaccination may be imposed in relation to specific population groups but not to the entire population, without exception. Some characteristic examples could be health care professionals, care home residents, those working in the food industry, and so on. In this respect, it would be decisive for the legislator to predetermine these specific population groups and, in doing so, the guiding principle would be to achieve the proper administration of justice, particularly when it comes to key medical professions. In the context of a hospital, for instance, a vaccinated member of staff should not have to take on all the hard work when unvaccinated members of staff would be able to put forward non-vaccination to avoid having to provide care to patients. Additionally, mandatory vaccination could assume an indirect nature through the acknowledgement of previous normality for vaccinated persons and the setting of restrictions for the unvaccinated. For instance, it will be easier for vaccinated persons to enter public transport, theaters, cinemas, gyms and so on, whereas the unvaccinated will have to present a specific medical examination. It should be noted at this point that there is often talk about the awarding of privileges, whereas it is submitted that a more accurate term of description would be ‘acknowledgement of a previous normality’, rather than ‘privilege’. Finally, it is of imperative importance to thoroughly inform the public regarding vaccination, as well as preclude any kind of confusion and misinformation in public opinion.

Considering the serious risks posed by COVID-19 to the health of individuals but also vis-a-vis public health, the essential constitutional question is not whether generally imposed mandatory vaccination is constitutionally permissible, but whether it is, in fact, constitutionally necessary. In this sense, what must also be considered is whether the omission on the part of the state to impose targeted mandatory vaccination where it is required [40] may be deemed unconstitutional in certain cases. This is the case because vaccination is one of the most valuable tools for public health protection and, as such, it also constitutes a positive obligation on the part of the state for addressing serious infectious diseases [39] (p. 245 et seq.).

## 5. The Case of Mandatory Vaccination at the Workplace

The first case of mandatory vaccination in Europe was in Italy and it concerned compulsory vaccination against COVID-19 for healthcare professionals and operators. Pursuant to Law Decree No. 44 (1 April 2021, Urgent measures to contain the COVID-19 epidemic, in the field of anti-SARS-CoV-2 vaccinations, justice and public competitions [41]), the Italian government introduced a vaccination obligation for health professionals. Those who refused to become vaccinated were able to choose to be assigned to tasks not implying risks of spreading the contagion or to be put in suspension, without remuneration, for up to one year. The Decree in question was met with the support of the Order of Doctors, Surgeons and Orthodontists (FNOMCeO). According to estimates, only 1 in 10.000 healthcare professionals still refuses to become vaccinated, whereas employees in health structures and care homes who have a lower level of expertise are those who seem to be more hesitant towards vaccination [42]. At the same time, the Decree in question has also been criticized for not introducing anything genuinely new [43]: on the basis of Articles 42 and 279 of the previous relevant Decree 81/2008, when specific biological risks are in existence, employers may either provide vaccines to employees or temporarily remove them from exposed activities (and transfer them to other positions, whenever such availability exists).

The position adopted by Great Britain’s Equality and Human Rights Commission on the question of mandatory vaccination is also along the same lines. According to the Commission’s relevant report, mandatory vaccination against COVID-19 for care home workers is a reasonable and justified measure, as the state is “right to prioritize protection of the right to life for residents and staff” [44]. Later on, the requirement for mandatory vaccination was also put in place in France [45].

It is reasonable to wonder whether a doctor or nurse has the right not to become vaccinated. The same line of questioning is also valid in relation to persons working in shops or health regulated establishments, such as cooks and serving staff, as well as persons working as drivers in public transport [24]. Beyond the flat refusal to become vaccinated and the corresponding moral condemnation of this refusal [46] in relation to certain occupations for which vaccination is imperative, the question that also arises is whether vaccination in the workplace could be rendered constitutionally mandatory.

Making vaccination in the workplace mandatory ultimately entails two prerequisites: Firstly, that the imposition of mandatory vaccination must be related to the nature of the work; and, secondly, the safety of the vaccine [47].

The reasonable question that arises in this context is whether an employer may impose a sanction to an employee who refuses to become vaccinated. It is submitted that for certain categories of employees, and under the circumstances of a pandemic, the imposition of a sanction is not excessive, but it should nevertheless be examined on the basis of the principle of proportionality. For example, the case of a doctor working in an Intensive Care Unit will be assessed differently than that of a business consultant who can also provide services remotely through teleworking, as will the case of a person working in a care home and that of a lawyer. It would be hoped that everyone had the conscientiousness to become vaccinated; however, the level and emergency character of mandatory vaccination could not have been the same [34].

Considering the above, it would be best if the legislator would proceed to determine in which cases vaccination will be mandatory, following the provision of detailed information to the public, instead of leaving the matter of the imposition of mandatory vaccination to the discretion of each individual employer who may be able to cite vaccination as a competitive advantage against competitors. What should also be highlighted is that in such a socially and morally controversial issue, such as mandatory vaccination, that divides society, and the answer to which is mostly the outcome of medical, philosophical, ideological and also financial perceptions, rather than one of purely legal argumentation based on the letter of the Constitution, the priority of decision making is vested primarily with the democratically elected legislator. Accordingly, the authority of interpretation held by the judiciary must be confined to the control of the outer limits of legislative choice [48] (p. 84). Judges ought to exercise self-restraint as there is always a risk of making an ideological and political choice of the democratically elected legislator into a “constitutional necessity or an excluded choice by constitutional requirement” [49] (p. 578).

In the case of Greece, the judiciary were called to provide an answer. An employee at a care home who refused to become vaccinated was fired. The answer on the part of the judge is not self-explanatory, considering that no relevant legislative provisions were in place at the time of the dismissal. At the instance in question, vaccination was not covered by the employment agreement; nevertheless, what could be examined was whether precluding the spreading of the disease in every way possible by someone who does not object to vaccination for health reasons could be considered an ancillary duty under the employment contract [50]. If vaccination, meaning the observance of measures for the protection of health, falls under one of the above duties, an employer exercising directorial duties is allowed to place the employee in question to a different position, amend related duties, put him or her on leave, and so on [50]. Notwithstanding the above, these options are not always forthcoming. A doctor who refuses to become vaccinated does not have an alternative choice but to come into contact with patients and cannot be transferred to an administrative service that does not entail contact with the public. Being unable to provide medical services to patients means that his or her vaccinated colleagues will be further burdened [51]. At the same time, the transferal to a different position that will not be dangerous in terms of disease transmission is not always the best choice. When an employer employs a surgeon to conduct surgeries and then this person cannot perform these duties due to the risk of spreading a disease, the employer is deprived of a very important employee due to the absence of vaccination, who would potentially not be useful if transferred to an administrative post. In view of the above, refusal to become vaccinated will cause problems in the functioning of the employer’s business, who may raise an objection for non-performance of the contract, pursuant to Article 374 of the Civil Code, and refuse to pay the related salary for as long as the employee fails to carry out his or her duties properly [52] (p. 711 and p. 743). At this point, it should be noted that the doctor in question had been employed to provide medical, rather than administrative, services. Placing a doctor to an administrative post gives rise to matters pertaining to the suitability of the employee, as he had not been previously considered for or had a trial period in this area [47]. The fact that resources for a certain service are finite and positions are specific is also of crucial importance, whereas the need to maintain sufficient staff for the fulfilment of the main mission of the service is also vital [47].

The prior substantiated advice on the part of the occupational doctor regarding the necessity of vaccination, as well as the observation of all necessary health and hygiene measures, is also of decisive importance [50]. Moreover, the employer must prove that the employee cannot offer the required services in a different way (for example, a physiotherapist cannot work remotely) and that the vaccine could sufficiently guarantee the protection of the health of the remaining employees at the workplace in question or those who visit it, and not only the health of the person to be vaccinated [50].

The selection of certain professions for which mandatory vaccination will be imposed must be conducted following careful planning. Emphasis is given to the medical profession due to its nature. In the case at hand, the medical profession creates special duties of trust and major diligence obligations [47]. Vaccinated health care professionals may impose less risk of disease transmission to patients, as documented in other infectious disease contexts including influenza and hepatitis B [53]. Health care professionals have chosen a particularly demanding profession and have given an oath to provide treatment, where an integral part of such treatment is the non-transmission of a disease [47]. They have a responsibility not to harm their patients [54]. Furthermore, the fact that patients themselves do not have a choice of doctor or rescuer at the crucial instance of emergency hospitalization should also not be overlooked [47]. When a patient is rushed into hospital on an emergency basis, there is no option of choosing a doctor who has been vaccinated. Therefore, the optimum solution must be provided for by the state.

Another question that arises is whether mandatory vaccination should be established solely on the basis of legislation or whether it could be a product of self-regulation. For example, can an employer impose it in the context of the exercise of directorial duties if the legislator has not selected it for a particular working field? It is submitted that employers may encourage, ask and convince employees to become vaccinated by example, but they cannot impose it by way of sanctions. This is because the privatization of public health policy bears the risk of unfair discrimination and, generally, of the division of populations into vaccinated and unvaccinated persons [55] (p. 674). The decision on mandatory vaccination and the related conditions is solely vested in the state. This is what is stipulated by Article 25§1 of the Constitution, according to which restrictions of any kind to fundamental rights “should be provided either directly by the Constitution or by statute, should a reservation exist in the latter’s favour, and should respect the principle of proportionality” [33].

## 6. Mandatory Vaccination in the Sectors of Education and Leisure

During the pandemic, children were called to carry a heavy burden towards its interception. They were confined to their homes and distance learning, having to put their zest for life temporarily on hold [56] (p. 46). The reason they were secluded was primarily to protect those vulnerable groups with which they would come into contact and, on a second level, in order to protect their own health. Distance learning was a temporary measure [57] that tested the limits of pupils’ patience, as well as that of their parents. It was imperative to find a solution that would allow pupils to gradually return to school, which is, after all their natural learning habitat. At the same time, any such solution would have to go hand in hand with public health protection; hence, this return did not take place heedlessly but materialized at a time when vaccination of vulnerable groups had already advanced. With the contribution of science, pupils had to be tested regularly to reduce, to the extent possible, the spreading of the virus. Following this, once the vaccine was approved for children over the age of 12, it also became a useful tool for the reopening of schools.

The dilemma relating to mandatory vaccination has entered the long-suffering sector of education. This particular question must be dealt with in a different way with reference to primary and secondary education, on the one hand, and the vast field of higher education, on the other hand. The necessity of having schools operate on-site is a given. At the time when vaccination had not been widely extended, restricting the right to education through the conduction of remote teaching was constitutionally tolerable [58] (p. 292 et seq.). The extension of vaccination, however, changes the relevant parameters and makes the smooth functioning of schools imperative. This aim can be achieved through extended vaccination. In schools where classes are smaller, control measures can be put in place. Mandatory vaccination for non-adults should be the last resort and puts many issues of constitutionality to question, as there is a lack of large-scale studies on the consequences of vaccination on minors. It is sensible that vaccination be recommended, and highly so, but it should not be imposed at this time. At the same time, it would also be reasonable that the sector of education should function in a controlled manner as regards the transmission of the virus. In this respect, the presentation of a self-test result by those pupils who do not wish to become vaccinated is a constitutionally tolerable choice. Nonetheless, self-testing bears the risk of non-safe certification and, thus, it should be examined whether it constitutes a right choice. To address this matter, one option that could be considered is the conduction of self-tests at school premises, obviously under conditions that will safeguard the protection of the personal data of pupils. In any event, the provision of free self-tests to pupils is certainly a step in the right direction.

Continuing to give lectures on-site at universities is set out to be a difficult challenge. Students in higher education are adults and vaccination may be made mandatory for them to enable them to attend lectures without putting public health at risk. The alternative option would be to present a negative test result. The issue with this option is whether this test result should be based on a self-test or an antigen one. The former is provided free of charge, whereas the latter is not. The second test could also potentially be offered free of charge to higher education students; however, this would be a matter of economic policy, as it is not feasible to offer all services for free. Furthermore, young people over 18 years of age must also be convinced to become vaccinated. It could be said that the alternative option of requiring that students be tested is coercive in a way, as students who lack financial resources may be forced to become vaccinated. Another possibility would be to allow students to attend classes remotely, through teleconference. Nevertheless, apart from any technical issues that may arise, this option is also very likely to adversely affect on-site teaching, as it is assumed that it will be the preferred option for students who do not reside at the location where their university is situated. For large and non-ventilated classrooms, however, it is advisable to put in place alternative modes of teaching.

Another question put forward is whether vaccination should be extended beyond the workplace to the leisure sector, such as, for example, to food services, tourism, sport, cultural activities, etc. It may also be said that it is not of vital importance that the leisure sector should continue to operate during the period of the pandemic or that restricting access solely and exclusively to vaccinated persons and those who had previously contracted the virus is excessive. The answer to the above position would be that leisure is of decisive significance for the continuation of cultural and economic activities, in a way that would be compatible with public health protecting. One way of achieving this is by permitting entry to indoors spaces only to vaccinated persons. An alternative possibility that could be considered is that of also allowing entry to those who can present a negative test result certificate.

## 7. Epilogue

The time has come to exculpate the discourse on mandatory vaccination [59]. As it transpires from the above ethical-legal analysis, mandatory vaccination conducted under certain conditions does not violate fundamental European legal. On the contrary, provided that the principle of proportionality is observed, mandatory vaccination constitutes a manifestation of the state’s obligation to protect the constitutionally enshrined fundamental rights to life, by proceeding to medical interventions that are permitted by the Constitution itself [60]. In this context, the importance of vaccination for the continuation of economic, social and cultural activities should also not be overlooked, as these are rights that are also guaranteed on the basis of relevant constitutional provisions. Making vaccination mandatory does not mean that it will be imposed by physical force, as is the case in non-democratic regimes such as China [61]; it will not turn into a scenario where someone will be after us with a needle to administer the vaccine, whether we want this or not. Nevertheless, it is reasonable that those who will choose not to become vaccinated, and who form part of a social group, may suffer certain consequences or, better still, that it would only be fair if those who do become vaccinated will regain the previously existing state of freedom more swiftly. Unvaccinated individuals, by analogy, will have to wait for the phasing out of the pandemic to return back their prior freedoms, for the sake of protecting society as a whole.

Those who do not wish to become vaccinated or disclose the sensitive personal data pertaining to (non) vaccination may freely make this choice [31]. Bearing in mind, however, that we all coexist in an organized society where, unfortunately, it appears that it will continue to be put at risk due to the emergence of various dangerous and infectious viruses and pandemics of various types and mutations. Therefore, it is tolerable that refusing to become vaccinated, which essentially equals refusal to fulfil the duty of social solidarity, may entail specific legal consequences [31].

The main conditions under which mandatory vaccination could be deemed acceptable are the following:

(a) The principle of proportionality must be observed, with mandatory vaccination being a last resort solution, following the exhaustion of milder measures, such as that of informing the public and attempting to persuade people to become vaccinated.
(b) In view of the fact that it constitutes a limitation to the right to self-determination, mandatory vaccination should be stipulated by law and be sufficiently specified with reference to each case where it is to be applied.
(c) It must be imposed following substantiated medical analyses, conducted under specific objective conditions.
(d) It must be temporally specified for the short period of the pandemic.
(e) It must not be associated with coercion [62].
(f) Its nature should be mostly that of an incentive, rather than a sanction [39,63] (p. 260).
(g) It should be preceded by the provision of detailed information to the population, coupled with an effort to preclude any kind of confusion and misinformation.
(h) It is self-evident that there will be availability of vaccines [39] (p. 260).
(i) An exception for purely medical reasons should be in place.

## Data Availability

Not applicable.
